# An inverse relationship between dental fluorosis and Molar Incisor Hypomineralization in Mexican schoolchildren in an area with a high concentration of fluoride in drinking water: A cross-sectional study

**DOI:** 10.1371/journal.pone.0310420

**Published:** 2024-09-16

**Authors:** Andrea Fernanda Medina Varela, Alvaro García Pérez, Teresa Villanueva Gutiérrez, Karen Angelina Mora Navarrete, Martha Patricia Nieto Sánchez

**Affiliations:** 1 Pediatric Stomatology Specialties, Faculty of Higher Studies (FES) Iztacala, National Autonomous University of Mexico (UNAM), Mexico City, Mexico; 2 Laboratory of Public Health Research, Faculty of Higher Studies (FES), Iztacala, National Autonomous University of Mexico (UNAM), Mexico City, Mexico; 3 Health Care Department, Metropolitan Autonomous University-Xochimilco, Mexico City, Mexico; Medical University of South Carolina, UNITED STATES OF AMERICA

## Abstract

**Aim:**

To evaluate the association between the frequency and severity of dental fluorosis and Molar Incisor Hypomineralization (MIH) in 8-12-year-old schoolchildren living in an area with a high concentration of fluoride in the drinking water.

**Methods:**

The present cross‑sectional study was conducted on Mexican children (n = 573) selected from one community presenting a drinking water fluoride concentration of 1.39 ppm/F. The prevalence of dental fluorosis was ascertained using the Thylstrup and Fejerskov Index (TFI). The presence and severity of MIH was evaluated using the European Academy of Pediatric Dentistry (EAPD) criteria. A multinomial regression model was used to estimate the odds ratio (OR) and the 95% confidence intervals (CI), using the severity of MIH as the result.

**Results:**

The prevalence of MIH was 37.7% and, by severity, was 16.1% mild, 14.3% moderate, and 7.3% severe. The prevalence of dental fluorosis in permanent dentition was 70.9% (TFI ≥1) and, by severity, was 29.2% (TFI = 0), 45.6% (TFI 1–3) and 25.3% (TFI ≥4), while 54.5% of subjects were found to have poor oral hygiene. Schoolchildren with fluorosis (TFI ≥4) were 49% less likely [OR = 0.51; p = 0.025] to present mild MIH than children with fluorosis (TFI <4). Similarly, children with fluorosis (TFI ≥4) were 53% [OR = 0.47; p = 0.019] and 62% [OR = 0.38; p = 0.036] less likely to present moderate and severe MIH than children with fluorosis (TFI <4).

**Conclusion:**

An inverse relationship between the presence of fluorosis and MIH was found. The results obtained by the present study may contribute to both the early identification of disorders affecting the enamel and the creation and implementation of long-term oral health prevention, promotion, and intervention programs in the affected population.

## Introduction

Dental development comprises a set of complex, multifactorial, and multi-level processes fundamentally controlled by the genome [1]. Developmental defects of enamel (DDE) are caused by complex interactions among the genetic, epigenetic, and environmental factors that play an indispensable role during amelogenesis; therefore, the type of defect depends on the exposure time corresponding to these factors during tooth formation [2]. DDE can present as defects affecting both enamel quality (hypomineralization) and quantity (hypoplasia) via the modification of or damage to the enamel organ [3]. Clinically, DDE sometimes present aesthetic, sensitivity, wear, and erosion problems as well as making teeth more susceptible to caries [4].

Fluorides play a central role in the prevention and control of caries, further, to being recognized as the main factor responsible for the decrease in the prevalence of caries on a global level [5]. However, it has been observed that the excessive ingestion of fluoride may directly subject the ameloblast to stress, impacting the synthesis of the proteins responsible for the elimination of the organic matrix [6] and, thus, producing a hypomineralization known as dental fluorosis. This defect is characterized by white, opaque, discolored, symmetrical, and diffuse stains and striations. In the most severe cases it can cause pigmentation, increased porosity, and the loss of enamel structure [7]. In Mexico, the prevalence of dental fluorosis ranges from 15.0% to 82.0% in areas with low/optimal levels of fluoride (<1.5 ppmF) and from 92% to 100% in areas with higher levels of fluoride (>1.5ppmF) [8].

An enamel defect characterized by opaque areas that vary from white to brown in color, an asymmetric pattern, and post-eruptive fractures in the enamel structure, Molar Incisor Hypomineralization (MIH) can affect one or more first molars and permanent incisors [9]. Although the etiology of MIH is not currently clear, various systemic, genetic, and/or epigenetic factors may impact amelogenesis during the maturation phase of the enamel matrix, resulting in a considerable decrease in its mineral content [10, 11]. The literature reports confusing results on the relationship between dental fluorosis and MIH, wherein research evaluating the association between fluorosis and MIH in populations with fluoridated water has found lower percentages of MIH [12, 13], while studies conducted in Brazil found a relationship between the severity of MIH (slight or severe) and dental fluorosis [11]. Lastly, other studies have reported that the ingestion of fluoridated water does not increase the incidence of MIH [14, 15]. Research into the relationship between these two enamel defects would help to improve the diagnostic process, given that MIH can be confused with dental fluorosis, as both disorders show diffuse symmetric opacities and the loss of structure in the affected teeth [16]. For this reason, early diagnosis and preventive care are important for the successful treatment of DDE. The present study aimed to evaluate the association between the frequency and severity of dental fluorosis and MIH in 8-12-year-old schoolchildren living in an area with a high concentration of fluoride in the drinking water. The hypothesis proposed by the present study is that schoolchildren with dental fluorosis (TFI ≥4) will present a significant inverse association with the severity of MIH.

## Material and methods

The present study was carried out in adherence to with the guidelines of the Strengthening the Reporting of Observational Studies in Epidemiology (STROBE) statement. The cross-sectional study was conducted from July 01 to December 30, 2023. The research protocol was reviewed and approved by the Ethics Committee of the Iztacala Faculty of Higher Studies at the National Autonomous University of Mexico (CE/FESI/062023/1619). Both the leadership team of the primary schools sampled and the participants’ parents were informed of the protocol, with those parents who agreed to the participation of their children signing the informed consent form.

According to the 2020 Population and Household Census conducted by the *Instituto Nacional de Estadística y Geografía* (INEGI or National Institute of Statistics and Geography) in Mexico, the study population was selected from the 13,032 inhabitants of a community called San Pedro Apatlaco, in the municipality of Ayala in the state of Morelos. In terms of social deprivation indicators, 26.9% of respondents had no access to food, 15.1% had no access to healthcare services, 26.8% had no access to basic household services (drinking water, sewage, and electricity), 20.7% presented educational lag, and 53.3% were living in moderate to extreme poverty [17]. The fluoride concentration in the drinking water was determined using a specific electrode (Thermo Scientific Orion Star ™, Waltham, MA, USA), with the samples analyzed according to the Official Mexican Standard (NMX-AA-077-SCFI-2001). The fluoride level of the drinking water in the study area was found to be 1.39 ppm/F [18].

The present study invited 720 schoolchildren currently enrolled in two public primary schools in the selected community, in which, it should be noted, there are no private schools. The *inclusion criteria* for the study were as follows: schoolchildren aged 8 to 12 years and of either gender; the four upper and lower incisors and the first four permanent molars fully erupted; and the parents/guardians of the participant residing at the same address. The *exclusion criteria* were as follows: schoolchildren who used a fixed orthodontic appliance; tooth malformation (amelogenesis imperfecta, dentinogenesis imperfecta, and dentinal dysplasia); and a history of dental trauma. In total, 95 of the schoolchildren did not present erupted first molars, 15 decided not to participate in the study, and the parents of 37 did not provide their signed informed consent. Therefore, once the eligibility criteria had been applied, the final sample for the study corresponded to 573 schoolchildren.

### Independent variable: Dental fluorosis

Dental fluorosis was evaluated using the TFI, with the buccal, occlusal, and lingual surfaces of the erupted permanent teeth examined. The TFI categories ranged from 0–9 and were developed based on the histological changes produced by different degrees of dental fluorosis [19]. The TFI index was chosen because it examines all teeth presents. The TFI was dichotomized into TFI = 0, TFI 1–3, and TFI ≥4. The coding was conducted based on the two teeth presenting the most severe fluorosis, with scores corresponding to categories 4 and higher (TFI ≥4) used as the cutoff value, thus including children with moderate to severe fluorosis.

### Dependent variable: MIH

The evaluation of MIH included the examination of the vestibular, occlusal/incisal, and palatal surfaces of all the permanent erupted molars and incisors, which were classified according to the criteria set out by the European Academy of Paediatric Dentistry (EAPD) [20]. Based on the EAPD index, mild MIH was present when demarcated enamel opacities were observed without the posteruptive loss of enamel and with only mild aesthetic concerns. The index classifies moderate MIH as the following: one yellow or brown demarcated opacity affecting less than one third of the tooth surface; two or more white or creamy demarcated opacities affecting at least one third but less than two thirds of the tooth surface; post-eruptive enamel breakdown ≤2 mm in diameter; and/or atypical restorations involving at least one third but less than two thirds of the affected tooth surface. Severe MIH was considered when, in addition to demarcated opacities, posteruptive enamel breakdown and persistent/spontaneous hypersensitivity affecting function were observed. The severity of MIH in each child was defined by the most severe defect observed in the first permanent molars or permanent incisors [4, 21].

### Covariables

The present study used the following variables as potential confounders: age (in years); gender (boy/girl); and toothbrushing frequency (number of times a day) dichotomized into < 2 or ≥ 2 times a day. the Simplified Oral Hygiene Index (OHI-S), which has two components—the debris and calculus indices, were selected from four posterior and two anterior teeth, with the results of the evaluation dichotomized into poor and good hygiene [22].

### Clinical oral examination

The oral examinations were performed in each school selected, using a mouth mirror and a WHO probe. The child was asked to brush their teeth prior to the examination to remove plaque or food remnants. The examinations were carried out with the assistance of a notetaker. The two examiners had been previously trained and calibrated, while the calibration process was divided into two phases (theoretical and clinical) for both MIH and dental fluorosis. The examiners inter and intra-examiner agreement corresponded to Kappa = 0.87 and Kappa = 0.89 for MIH and dental fluorosis, respectively.

### Sample size

The sample size was calculated using the formula for two independent proportions with 80% power, while a 0.12 difference in proportion was detected between the two groups, with a bilateral p value of 0.05. Assuming that 23% of the participants, as selected from the population of reference, present the factor of interest (MIH), the present study required a sample size of 237 per group, namely a total sample size of 474, assuming equal-sized groups [23].

### Statistical analysis

All the statistical analysis was carried out using the Stata 15 program (Stata Corp, College Station, TX, USA). Xi-squared tests were used to determine the associations among the variables of gender, oral hygiene, toothbrushing frequency, dental fluorosis, and MIH. Multinomial regression was used to analyze the association between the independent variable dental fluorosis (TFI<4 = 0 and TFI ≥4 = 1) and the dependent variable MIH (mild, moderate, and severe), controlling for covariates such as gender, age, and oral hygiene, which was expressed as an odds ratio (OR) with 95% confidence intervals (CI). In all the analysis conducted, two-tailed values of p<0.05 were considered statistically significant.

## Results

### Characteristics of the study population

**Fig 1:**
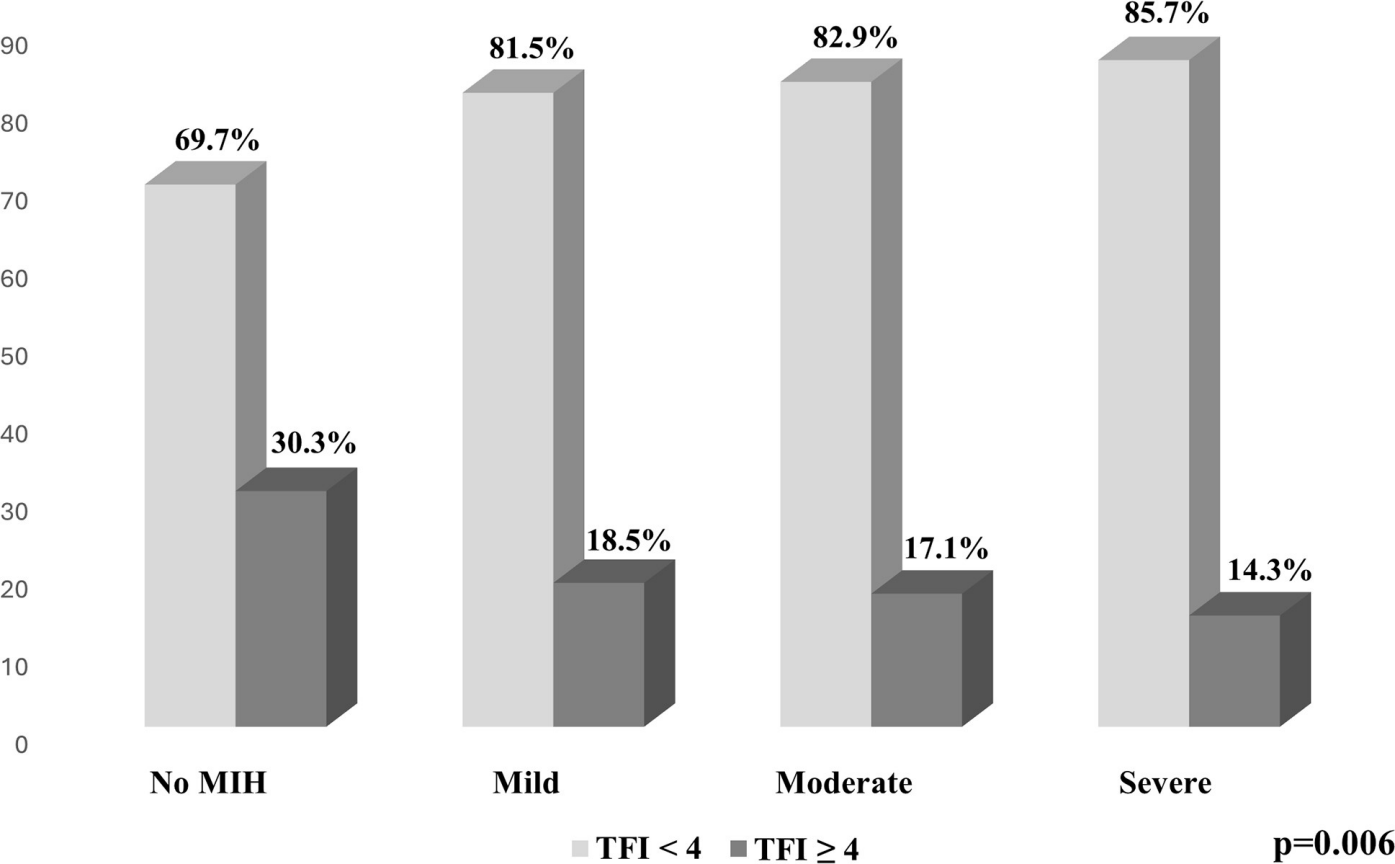
Percent distribution between dental fluorosis and Molar Incisor Hypomineralization (MIH) among Mexican schoolchildren.

A total of 573 schoolchildren aged 8–12 years, with a mean age of 9.32 (± 0.91), were included in the present study, while the percentages of boys and girls examined were 51.7% and 48.3%, respectively, with no significant differences found between the mean age by gender (p = 0.885). The findings obtained reveal that 46.8% of the schoolchildren had poor oral hygiene. The prevalence of MIH was 37.7%, while the distribution of MIH severity among the affected schoolchildren was 16.1% mild, 14.3% moderate, and 7.3% severe. The prevalence of dental fluorosis in permanent dentition was 70.9% (TFI≥1), and, by severity, 29.2% (TFI = 0), 45.6% (TFI 1–3), and 25.3% (TFI ≥4). Table 1 presents the characteristics observed in the sample in schoolchildren both with and without MIH, with a higher proportion of girls than boys presenting MIH (50.9% vs 49.1%), although the difference was not found to be statistically significant. Participants with fluorosis (TFI ≥4) presented a lower proportion of MIH than participants with fluorosis (TFI < 4) (17.1% vs 82.9%), with a significant association observed between both variables (p<0.001). Fig 1 presents an inverse relationship between the presence of fluorosis (TFI ≥4) and the severity of MIH, where it was observed that those schoolchildren with fluorosis (TFI ≥4) presented a lower severity of MIH in its three categories (mild, moderate and severe), thus revealing a significant relationship (p = 0.006).

**Table 1:** Demographic and clinical characteristics in schoolchildren with and without Molar-Incisor Hypomineralization (MIH) (n = 573).

	No MIH n = 357 n (%)	MIH n = 216 n (%)	p*
**Age (Mean, SD)**	9.32 (±0.89)	9.34 (0.95)	0.795
**Gender**			
Boys	190 (53.2)	106 (49.1)	0.336
Girls	167 (46.8)	110 (50.9)	
**Oral hygiene (OHI-S)**			
Good hygiene	195 (54.6)	110 (50.9)	0.390
Poor hygiene	162 (45.4)	106 (49.1)	
**Toothbrushing frequency**			
< 2 times a day	192 (53.8)	113 (52.3)	0.733
≥ 2 times a day	165 (46.2)	103 (47.7)	
**MIH degree**			
No MIH	357 (100.0)	0 (0.0)	-
Mild	-	92 (42.6)	
Moderate	-	82 (37.9)	
Severe	-	42 (19.5)	
**Dental fluorosis (TFI)**			
TFI < 4	249 (69.7)	179 (82.9)	<0.001
TFI ≥ 4	108 (30.3)	37 (17.1)	

*Chi-square test

The results of the multinomial logistic regression analysis applied showed that the presence of dental fluorosis (TFI ≥4) was associated negatively with the severity of MIH after adjusting for age, gender, and oral hygiene, with the corresponding results shown in Table 2. The participants with fluorosis (TFI ≥4) were 49% less likely [OR = 0.51 (0.29–0.92); p = 0.025] to present slight MIH than the children with fluorosis (TFI <4). Similarly, participants with fluorosis (TFI ≥4) were 53% [OR = 0.47 (0.25–0.87); p = 0.019] and 62% [OR = 0.38 (0.15–0.93); p = 0.036] less likely to present moderate and severe MIH than those with fluorosis (TFI <4). The variables age, gender and oral hygiene were not significant in the regression model.

**Table 2:** Multinomial logistic regression model for the association between dental fluorosis (TFI) and Molar-Incisor Hypomineralization (MIH) among Mexican schoolchildren (n = 573).

		Molar-Incisor Hypomineralization		
		Mild	Moderate	Severe
Variables		Odds Ratio (95%CI)	Odds Ratio (95%CI)	Odds Ratio (95%CI)
**Gender**	Boys	*Reference*	*Reference*	*Reference*
	Girls	1.10 (0.69–1.75) p = 0.670	1.21 (0.75–1.97) p = 0.415	1.40 (0.73–2.68) p = 0.300
**Age**		0.95 (0.73–1.22) p = 0.694	1.08 (0.83–1.40) p = 0.550	1.06 (0.74–1.51) p = 0.728
**Oral Hygiene (OHI-S)**	Good hygiene	*Reference*	*Reference*	*Reference*
	Poor hygiene	1.14 (0.71–1.82) p = 0.572	1.22 (0.75–2.00) p = 0.405	1.12 (0.58–2.14) p = 0.729
**Dental fluorosis (TFI)**	TFI < 4	*Reference*	*Reference*	*Reference*
	TFI ≥ 4	0.51 (0.29–0.92) **p = 0.025**	0.47 (0.25–0.87) **p = 0.019**	0.38 (0.15–0.93) **p = 0.036**

CI = Confidence Interval.

## Discussion

The present study found that Mexican schoolchildren with fluorosis (TFI ≥4) are less likely to present moderate or severe MIH than their peers with fluorosis at degrees of TFI <4. Few studies have reported inverse results for the association between dental fluorosis and the severity of MIH.

Zhang *et al*., in schoolchildren in China showed that MIH was strongly negatively correlated with dental fluorosis, as the severity of MIH decreased, the incidence and severity of dental fluorosis decreased [16]. Similarly, Restrepo *et al*., in adolescents aged 13–16 years, found that the frequency and severity of MIH tend to be lower in the presence of dental fluorosis (OR = 0.02) [24]. Meanwhile, Fernandes *et al*. found, in Brazilian schoolchildren aged 6–12 years, that dental fluorosis was associated with the presence of severe MIH (OR = 4.46) in areas with moderate to high concentrations of fluoride in their drinking water [11].

While low concentrations of fluoride help to protect and strengthen the enamel, fluoride, at high concentrations, can damage the ameloblast [25]. Various studies have shown that fluoride negatively affects the ameloblasts in various cellular functions, inhibiting the synthesis and secretion of proteins and cell cycle progression [26], causing oxidative stress, and damaging DNA [27]. Another important aspect to consider is the effect of fluoride on calcium homeostasis and the endoplasmic reticulum [28]. High concentrations of fluoride coming into contact with the endoplasmic reticulum reduces calcium levels and induces a continual state of stress in the endoplasmic reticulum while storing the calcium, an effect reflected in the formation of enamel crystals, which impacts mineralization [29]. Generally, while the defects that present during the maturation phase result in a normal volume of enamel, they also lead to insufficient mineralization [30]. One of the most notable characteristics of enamel damaged by MIH is the actual amount of organic material that it presents compared to healthy enamel. In a systematic review, Elhennawy *et al*. found that hypomineralized enamel presents lower Ca and P concentrations, lower hardness, a lower modulus of elasticity, higher concentrations of carbon and carbonate, and a higher protein content (10–20 times) than healthy enamel. As a result, enamel damaged by MIH presents greater porosity, more cracks in the tooth, and deep pores [31]. In other terms, enamel diagnosed with fluorosis and MIH may not have completed the maturation stage during amelogenesis; therefore, a stronger association between fluorosis and MIH presents in areas where fluorosis with levels of TFI ≥4 occurs with greater frequency [11]. Although other factors have been reported to be related to the development of MIH [32], the present study found that the severity of MIH reduced in the presence of fluorosis (TFI ≥4). To date, no conclusive evidence is available in the literature on the association between dental fluorosis and MIH, prompting a great need for future epidemiological and experimental research to evaluate the role played by fluoride in the etiology of MIH.

On an international level, the prevalence data for MIH reported by the few studies conducted in areas with different fluoride concentrations in the drinking water are heterogenous and range from 7.6% to 38.6% in countries such as Dubai, India, Brazil, and Mexico [11, 33–35]. The present study observed a 37.7% prevalence level for MIH, which is consistent with previous findings observed in Mexican children [21, 36, 37]. The differences found among these studies on the occurrence of MIH in areas with different fluoride concentrations in the drinking water can be attributed to the sample size, the age groups sampled, the diagnostic criteria used, and the use of different indices for evaluating MIH.

The present study found, in the community selected for the research, a fluoride concentration of 1.39 ppm/F in the drinking water and a 25.3% prevalence of dental fluorosis, which was found to impact the appearance of the entire tooth surface (TFI ≥4). Various studies have used the TFI to evaluate the prevalence of fluorosis [38, 39]. In Mexican schoolchildren living in areas with fluoride concentrations of 1.01mg/L in its drinking water, Pérez-Pérez *et al*. found a prevalence of fluorosis of 95.7% (TFI >0), which fell to 45.2% (TFI ≥4) for the moderate and severe categories [40]. The data obtained is likely to result from exposure to multiple sources of fluoride, including fluoridated salt and water [41]. Therefore, a greater understanding of the risk factors related to dental fluorosis would help in the identification and creation of educational strategies able to prevent the high level of consumption of fluoride in populations presenting endemic fluorosis.

Finally, the present study did not find an association between poor oral hygiene and the presence of MIH. Various studies have indicated that the presence of fluorosis and moderate/severe MIH creates conditions more susceptible to the occurrence of caries, given the loss of enamel continuity and the fractures that facilitate the accumulation of biofilm on the enamel, thus exposing the dentine [42]. Therefore, increasing toothbrushing frequency in schoolchildren presenting fluorosis and MIH would assist in reducing the accumulation of biofilm, halitosis, and the formation of initial carious lesions.

One of the limitations of the present study was its cross-sectional design, which meant that it was not possible to establish cause-effect relationships. However, the data collection carried out was standardized, including two trained examiners with experience in the use of the indices for MIH and dental fluorosis. Secondly, another limitation was the difficult differential diagnosis among MIH, fluorosis, and initial carious lesions, although this challenge only presented in a few of the participating schoolchildren due to the adequate calibration process undergone by the two examiners who collaborated in the present study.

## Conclusions

The present study found an elevated prevalence of fluorosis and MIH, furthermore, an inverse relationship was found between the presence of fluorosis and MIH. The results of the present study may contribute to the identification and early diagnosis of disorders affecting the enamel, as well as the creation and implementation of long-term oral health prevention, promotion, and intervention programs that aim to improve oral health conditions in schoolchildren.

## Supporting information

S1 File (XLSX)
